# Polygenic scores in psychiatric research and clinical practice

**DOI:** 10.1515/medgen-2026-3012

**Published:** 2026-07-08

**Authors:** Leonard Frach, Friederike S. David, Markus M. Nöthen, Andreas J. Forstner

**Affiliations:** University Hospital Bonn, University of Bonn Institute of Human Genetics Venusberg-Campus 1 53127 Bonn Germany; University of Bonn, University Hospital Bonn Institute of Human Genetics Venusberg-Campus 1 53127 Bonn Germany; University of Bonn, University Hospital Bonn Institute of Human Genetics Venusberg-Campus 1 53127 Bonn Germany; University of Bonn, University Hospital Bonn Institute of Human Genetics Venusberg-Campus 1 53127 Bonn Germany

**Keywords:** Biological subtypes, Genomics, PGS, Prediction, Psychiatric genetics

## Abstract

Polygenic scores are nowadays common tools in psychiatric genetic research and are also increasingly applied in clinical settings. In this article, we give an overview of polygenic scores in psychiatric research, focusing on recent trends and novel applications such as using polygenic scores in family studies. Furthermore, we discuss use cases of polygenic scores in clinical studies and clinical practice, for instance using polygenic scores to predict treatment response to medication. While some challenges of polygenic scores are not unique to psychiatry (e.g. limited reliability or transferability across ancestries), we also highlight the specialties of using polygenic scores in psychiatric research and clinical practice (e.g. clinical and biological heterogeneity across and within psychiatric conditions), as well as limitations of other applications beyond research, e.g. using polygenic scores for psychiatric conditions for embryo selection. We further make the case for careful communication of genetics to patients and the general public. We conclude by providing an outlook which includes future directions and applications of polygenic scores in psychiatric research and clinical practice.

## Introduction

Psychiatric conditions represent a major contributor to the global burden of disease [1]. Current therapies are suboptimal [2], and psychiatric patients have an increased mortality risk, e.g. due to death by suicide [3]. Psychiatric conditions encompass diverse disorders of varying prevalence. In Germany, for example, prevalences range from less than 1 % for schizophrenia [4] to 16 % for depression [5]. Typically, psychiatric conditions are multifactorial and involve a complex interplay between genetic and environmental factors [6]. Psychiatric conditions also vary in terms of childhood versus adult onset. For example, neurodevelopmental conditions, such as autism and attention deficit hyperactivity disorder (ADHD), typically present in early childhood, whereas a first diagnosis of depression is more common in adulthood. This is important as the relative contribution and the type of genetic factors influencing psychiatric conditions depend on the environmental context and can vary over time and development [7]. Similarly, both genetic and environmental factors influence trajectories of psychiatric conditions, e.g. persistence of symptoms across development [8].

**Figure 1:**
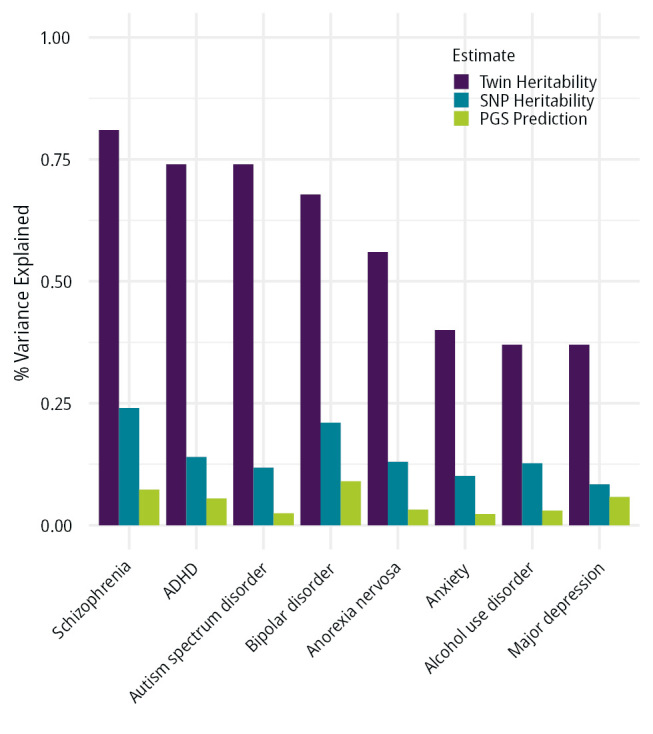
Heritability estimates and PGS prediction for psychiatric conditions *Note. ADHD = attention deficit hyperactivity disorder. Anxiety = anxiety disorders, including generalised anxiety disorder, panic disorder or phobias. Heritability estimates from twin studies* [12,37 – 42]* as well as single nucleotide polymorphism (SNP) heritability and PGS prediction estimates* [33, 43 – 50]* are taken from the referenced studies.*

Available research suggests that for many psychiatric conditions, the underlying genetic architecture is highly polygenic, with thousands of common genetic variants distributed across the genome each contributing small additive effects to disease liability [9, 10]. Most variants individually fall below genome-wide significance but jointly contribute substantially to SNP heritability and to the predictive performance of polygenic scores (PGS). In addition, numerous rare variants of more direct functional consequence further shape risk [11]. Importantly, heritability estimates (phenotypic variance explained by genetic factors) across psychiatric conditions vary between ~ 40 and ~ 80 % [12] (see **Figure 1**).

Cross-disorder variance is also observed in terms of the relative contribution of common and rare variants [11]. For instance, in exome sequencing studies of rare coding variants, ten disease genes were implicated for schizophrenia based on a study sample of 121,570 individuals [13], while for depression, only two disease genes were highlighted based on 320,356 individuals [14]. These observations highlight the genetic heterogeneity of psychiatric conditions both in terms of heritability, as well as in terms of the specific genetic factors involved (e.g. partly distinct genetic pathways and the role of rare variants in addition to common variants). In contrast, there is widespread pleiotropy between different psychiatric conditions, whereby genetic variants are associated with multiple phenotypes [15], which points towards some shared genetic aetiology of psychiatric conditions (e.g. similar genetic pathways involved in bipolar disorder and schizophrenia).

Pleiotropic effects of genetic variants on multiple conditions can be either concordant or discordant (antagonistic) in terms of effect direction. If pleiotropic effects on two conditions (say schizophrenia and bipolar disorder) are concordant, this can enhance cross-phenotype prediction, as a PGS for schizophrenia for example, can also be associated with an increased risk for bipolar disorder. At the same time, concordant pleiotropic effects may complicate the utility of PGS for the clinical stratification between disorders with correlated genetic signals. PGS and approaches to assess global genetic correlations, such as linkage disequilibrium score regression [16] depend on the direction of SNP effects, e.g. concordant directions of SNP effects between traits, with antagonistic effects decreasing the cross-trait association. Other methods like MiXeR [17] assess genetic overlap (pleiotropy) between conditions irrespective of effect direction. Together with local genetic correlations [18], these analyses can provide more insights in the shared genetic pathways between psychiatric conditions [19, 20]. For PGS prediction, information such as local genetic correlations between phenotypes can be integrated to enhance prediction [21].

In both clinical practice and research, psychiatric conditions are currently classified and diagnosed using the Diagnostic and Statistical Manual of Mental Disorders (DSM) [22] or the International Classification of Diseases (ICD) [23]. ICD and DSM are descriptive, symptom-based classification systems which categorise disorders on the basis of observed behaviour and reported subjective experiences (symptoms) rather than pathophysiological markers*.* In both systems, psychiatric conditions are grouped into specific, diagnostic subdomains on the basis of symptom clusters, aetiology, and developmental onset. For instance, the domain of mood disorders includes depressive and bipolar disorders, both involving depressive symptoms like depressed mood, loss of interest or pleasure in activities and reduced energy.

Importantly, both within and across domains, psychiatric conditions and the symptoms thereof often co-occur [24], impact one another [25, 26], and share common genetic [11] and environmental risk factors, with the latter including factors such as low socioeconomic status [27] and childhood trauma [28]. Furthermore, substantial clinical and genetic heterogeneity exists across individuals with the same clinical diagnosis [29 – 31]. This poses both challenges and opportunities for psychiatric research in general and for psychiatric genetics in particular. Specifically, genetic findings challenge current diagnostic categories which are not based on biological disease mechanisms [10, 32]. For instance, bipolar disorder type 1 (at least one manic episode) and type 2 (at least one hypomanic and depressive episode) are genetically somewhat dissimilar and show distinct genetic correlations with other conditions [33]. Specifically, bipolar disorder type 1 is genetically (and phenotypically) more similar to schizophrenia, whereas bipolar disorder type 2 is more closely related to depression [33]. This suggests that transdiagnostic approaches, including both clinical features as well as biomarkers such as genetic factors are warranted for both psychiatric research and clinical practice.

Polygenic scores, also termed polygenic risk scores in the context of adverse clinical outcomes (e.g. disease or disorder), are weighted sum scores of the additive genetic effects for a given trait or disorder. Typically, PGS are based on common variants and therefore only measure the component of genetic liability captured by common genetic variation. In addition, PGS are imperfect measures of this contribution of common variants, as well as of the total genetic contribution to a trait or disorder [34]. The increasing use of genome-wide association studies (GWAS) has rendered PGS more relevant, since PGS predictive power and accuracy increase with larger GWAS sample size [35]. Psychiatric genetics has been at the forefront of development of statistical genetics methods such as PGS [9] by early on harnessing collaboration through international consortia (e.g. the *Psychiatric Genomics Consortium*, PGC). PGS are now commonly applied in psychiatric genetic research, and their use in clinical practice is increasing with improved methods. The development of novel PGS methods is ongoing, for instance, to increase prediction or transportability across ancestries (see Maj, Klinkhammer, and Mayr (this issue) and ref [36] for an overview). Importantly, the predictive power of PGS depends on the heritability of the studied phenotype [35]. Hence in psychiatry, cross-diagnosis differences in PGS predictive power are inevitable.

Clinical application of PGS in psychiatry (e.g. informing diagnosis, treatment or prevention) requires solid evidence for practical benefits based on research. Whereas basic research is informed by statistically significant findings involving PGS, clinical application further demands added *clinical* significance of introducing PGS to current standard procedures in diagnostics and treatment. Here, we present recent advances in the use of PGS within psychiatric genetic research and early adoptions in clinical psychiatric practice. However, the boundaries between PGS applications in research and psychiatric clinic are blurred, as the ultimate goal in psychiatric genetics is to benefit patients by better understanding their conditions and thus improve treatment or prevention. Similarly, challenges of using PGS are equally relevant for both research and clinical applications, some of which include imperfect reliability of PGS and environmental confounders. As an example beyond application in psychiatric clinical practice, we briefly discuss scientific and ethical challenges of using PGS for psychiatric conditions for embryo selection. Finally, we point out the relevance of communication of genetics in psychiatry and conclude with an outlook on potential future developments in the field.

## Polygenic scores in psychiatric genetic research

Polygenic scores have been first introduced in 2009 [9] and have been used in hundreds of research studies on psychiatric genetics since. To date, psychiatric genetic researchers have applied PGS to address multiple issues. Here we highlight six use cases, including phenotype prediction, heterogeneity within clinical diagnoses, disease biology, developmental course, treatment outcomes and intergenerational risk transmission (see **Box 1**).

PGS can be used to predict the target phenotype (e.g. the psychiatric condition analysed in the respective GWAS) in independent samples, and are also frequently applied for cross-phenotype prediction, i.e. associating a PGS for bipolar disorder with genetically-related phenotypes such as depression or schizophrenia. Research has shown that PGS predict psychiatric disorders (e.g. schizophrenia or depression) independent of family history, and that PGS explain a substantial proportion of phenotypic variance [51, 52]. Albeit family history and PGS partially measure the same underlying genetic liability, they can be seen as complementary [53]. While family history likely captures additional risk from rare variants and shared environmental factors within the family, this binary information is prone to reporting error, e.g. when no information on family members is available. Conversely, PGS provide a continuous and more ‘objective’ measure of individual risk captured by common variants, which however is also influenced by measurement error [34, 54].

Box 1: Key applications of PGS in psychiatric research

**Prediction**: Research has shown that PGS predict psychiatric disorders (e.g. schizophrenia or depression), independent of family history.

**Underlying biology**: Understanding the biological mechanisms involved in psychiatric conditions can be improved using more biologically functional PGS, integrating other omics data or by examining cell-type specific effects of PGS.

**Clinical heterogeneity**: PGS can differentiate individuals with ADHD only from individuals with additional co-occurring conditions (e.g., autism spectrum disorder or substance use), and have generated insights into the clinical heterogeneity of psychiatric conditions (e.g. depression or bipolar disorder).

**Developmental course**: Associations between PGS and symptoms of psychiatric conditions vary across developmental stages and PGS can predict distinct trajectories of symptoms (e.g. depression symptoms from adolescence to adulthood).

**Treatment response**: PGS have been used to predict pharmacological treatment outcomes (e.g. response and remission) in individuals with mood and psychotic disorders.

**Intergenerational risk transmission**: PGS have been used to show direct and indirect genetic effects in the intergenerational transmission of psychiatric conditions (e.g. ADHD).

Understanding of the biological mechanisms involved in the aetiology of psychiatric conditions can be improved using more biologically functional PGS, such as pathway-based PGS [55] and transcriptome-based PGS [56], or by examining cell-type specific effects of PGS in the brain [57]. PGS have also been used to identify distinct biological pathways, i.e. pathways beyond those implicated in shared genetic effects between different psychiatric disorders. These analyses were performed using data from case-case GWAS that were designed to contrast the underlying genetic architecture of two different psychiatric disorders, such as major depression and bipolar disorder [58, 59].

In addition to biological heterogeneity across and within psychiatric conditions, PGS allow the investigation of clinical heterogeneity, e.g. by dividing psychiatric patients with a specific diagnosis into those who do and those who do not have other co-occurring psychiatric conditions. Differing polygenic profiles can inform about age of onset or symptom severity, e.g. with higher polygenic burden being associated with an earlier age of onset [60] and greater symptom severity [61]. For instance, PGS can differentiate individuals with ADHD only from individuals with additional co-occurring conditions (e.g., autism spectrum disorder or substance use) [62], and have generated insights into the clinical and genetic heterogeneity of depression [63], bipolar disorder [64], and autism [65].

**Figure 2:**
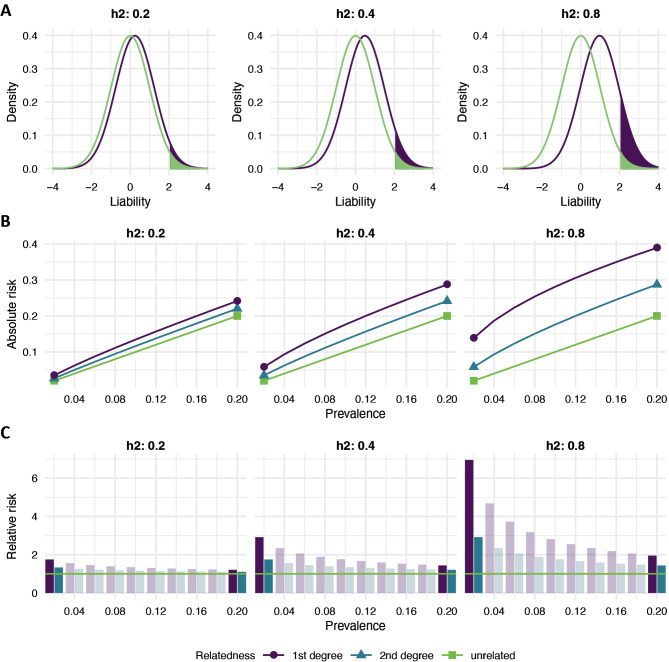
Absolute and relative risk for psychiatric conditions, based on prevalence, heritability, and familial risk (affected 1st or 2nd degree relative) *Note*. h2 = heritability. These estimates are based on heritability and not PGS prediction. However, (SNP) heritability estimates can be seen as the upper boundary of potential PGS prediction and PGS prediction can approximate heritability estimates for some phenotypes with large enough sample sizes [96]. Thus, these estimates are overly optimistic, especially for scenarios with high heritability (0.80), which is based on twin heritability estimates. **A** Liability threshold model showing as an example the liability (risk) for a disorder with a population prevalence of 2 % (e.g., comparable to bipolar disorder or panic disorder) for individuals with 1st degree affected family members vs. individuals with no affected family members. **B** Absolute risk estimates for different combinations of prevalence and heritability for individuals with no affected family members (green line) vs. individuals with affected 1st degree (purple line) or affected 2nd degree (blue line) relatives. **C** Relative risk for individuals with familial risk (affected 1st or 2nd degree relatives) compared to those without familial risk (set to 1, green line as baseline) for different prevalence and heritability combinations, based on Figure 2B. For calculations see https://github.com/LeonardFrach/PGS_in_psych.

Genetic models of psychiatric conditions typically assume an underlying liability threshold model, i.e. the genetic risk liability for a specific psychiatric condition is normally distributed and individuals passing a threshold of genetic risk will develop the respective condition (**Figure 2**). Importantly, besides influencing the liability for a psychiatric condition, genetic factors also influence age of onset, disease severity, and developmental course (i.e. progression of symptoms over time) and response to treatment. For instance, associations between PGS and psychiatric symptoms can vary across development, e.g. between childhood and adolescence [66]. Furthermore, PGS influence trajectories of psychiatric conditions, e.g. persistence of depressive symptoms from adolescence to adulthood [8]. In addition, PGS have been used to predict pharmacological treatment outcomes (e.g. response and remission) in individuals with mood and psychotic disorders [67 – 69]. For an overview, see [106].

In recent years, psychiatric genetic researchers have also used PGS to study intergenerational risk transmission. For example, the investigation of family trios allows the study of both direct genetic effects (of an individual’s genotype on their own phenotype) and indirect genetic effects. Indirect genetic effects are effects of an individual’s genotype on the phenotype of another individual; in family trios often from parent to offspring. Growing evidence suggests that indirect genetic effects are implicated in psychiatric conditions, particularly during childhood. For example, using this design, researchers have demonstrated both direct and parental indirect genetic effects on ADHD in early and late childhood [70, 71]. However, less clear evidence exists for indirect genetic effects on psychiatric conditions in later development, e.g. depression or anxiety disorders in adulthood [72]. Importantly, research suggests that indirect genetic effects can confound direct genetic effects. Therefore the use of unrelated individuals to assess genetic effects (e.g. using PGS) can result in an overestimation of direct genetic effects [73]. This is more relevant for social and behavioural phenotypes (including psychiatric conditions) than for somatic diseases.

Besides these key applications, PGS have also been used in psychiatric genetic analyses to address causal questions [74 – 77], whereby the potential causal effects of specific risk factors are tested using methods such as Mendelian randomisation.

## Polygenic scores in psychiatric clinical practice

Discussion of the translation of PGS into the (pre-)clinical psychiatric setting requires differentiation between distinct applications with varying levels of implementation readiness. Clinically relevant implementation of PGS can be distinguished into use cases i) at population level (e.g. screening and prevention), ii) in at-risk individuals (e.g. family members of individuals with psychiatric conditions) or iii) in clinical populations, i.e. already diagnosed patients. Population-level screening is currently unjustified, given modest absolute risks (e.g., 4 % – 6 % lifetime risk for top 1 % of schizophrenia PGS versus 1 % population prevalence, [78, 79]) and the lack of established preventive interventions.

More promising applications target at-risk individuals defined through family history and environmental risk factors, for whom integration of PGS may improve risk prediction [51, 52]. In these individuals, regular monitoring and early intervention strategies may prevent disease onset, or ameliorate disease course through timely access to effective treatments. Similarly, in patients presenting with prodromal or attenuated symptoms that do not yet meet full diagnostic criteria, PGS can contribute to risk stratification and inform decisions regarding monitoring intensity and early intervention. PGS may also refine risk assessment in carriers of rare high-penetrance variants for which psychiatric conditions are part of the phenotypic spectrum. In 22q11.2 deletion syndrome (22q11DS), the baseline risk of schizophrenia is approximately 20 – 25 % [80, 81], which is a 20 – 40-fold increase in risk compared to the general population. Schizophrenia PGS can be used to stratify this risk. For example, a study showed that when comparing extreme deciles, 33 % of 22q11DS carriers with high PGS developed schizophrenia compared to 9 % with low PGS [82].

Beyond risk prediction, PGS can facilitate the identification of biologically meaningful subtypes within heterogeneous diagnostic categories. Integration of PGS with other omics data (e.g. DNA methylation [83] or proteomics [84]) and neuroimaging [85] has been used to delineate potential subtypes characterised by distinct pathophysiological mechanisms. Examples include the immunometabolic subtype of major depression (characterised by elevated body mass index and C-reactive protein PGS, increased appetite, and weight gain) [86]. Current PGS lack sufficient discriminative ability as standalone diagnostic tools due to substantial cross-disorder genetic overlap [87]. However, they may make a meaningful contribution to clinical differentiation when integrated with detailed phenotyping and longitudinal assessment.

Preliminary evidence from studies of patients with major psychiatric disorders suggests associations between PGS and treatment resistance, disease course, and response to specific interventions (e.g. electroconvulsive therapy, lithium). However, PGS derived from case-control studies may not predict therapeutic outcomes in an optimal manner, since the developmental course of psychiatric conditions and treatment response probably involve partially distinct genetic architectures than the mere liability for these psychiatric conditions [88,89]. The development of treatment-specific PGS requires large, longitudinally assessed cohorts and standardised assessments of response [79, 87].

The most ethically contentious application concerns PGS-based embryo selection when using *in vitro* fertilisation. While prohibited in Germany under the Embryo Protection Act and Preimplantation Genetic Diagnosis Act (which restricts testing to high-risk monogenic disorders or genetic causes of miscarriage), increasingly, commercial PGS-based selection is being offered in other countries to prospective parents who seek to reduce risk for complex conditions. Beyond ethical concerns, which are detailed elsewhere [90,91], there are also some methodological concerns of using PGS for psychiatric conditions for selection [92]. Current predictive accuracy combined with limited embryo availability substantially constrains potential risk reduction. Moreover, PGS for psychiatric conditions and behavioural traits may be prone to genetic and environmental confounders [73], including effects of population stratification and indirect genetic effects (see above). This has important implications in terms of the potential utility of PGS for embryo selection, since selection can only mitigate risk conferred by direct genetic effects. In the case of gene-environment interactions, changing environmental conditions pose an additional challenge, as PGS-phenotype associations known at the time of embryo selection may not hold true over subsequent decades [93], which is particularly important for adult-onset psychiatric conditions. The widespread pleiotropy of psychiatric conditions also poses a challenge in terms of PGS-based embryo selection. For example, a PGS for schizophrenia could be associated with both a lower risk for schizophrenia and a lower risk for other psychiatric disorders, such as bipolar disorder. However, selecting based on the PGS for schizophrenia could lead to unintended side effects on other traits that are (genetically) correlated with schizophrenia, such as creativity [94] or language ability [95]. These effects cannot yet be comprehensively estimated, and require further investigation and careful consideration. In summary, numerous scientific and ethical concerns exist with regard to PGS-based embryo selection, and the spectre of eugenics demands proactive governance frameworks and inclusive international dialogue well in advance of technical feasibility and clinical utility.

## Communication to patients and the general public

Communication of genetic information presents particular challenges in psychiatry [97]. Genetic literacy, i.e. the capacity to understand and appropriately use genetic information, is limited in the general population and may be further compromised during acute psychiatric illness. Unlike monogenic disorders, polygenic contributions are probabilistic and context-dependent, and require nuanced interpretation. Research suggests that genetic information can influence symptom perception and wellbeing, and that individuals may retrospectively recall more symptoms when informed of high genetic risk [98]. In psychiatric populations, poorly communicated genetic information risks reinforcing fatalistic attitudes or undermining treatment engagement. Appropriately contextualised information, which emphasises modifiability, environmental contributions, and probabilistic nature, may reduce self-blame and facilitate shared decision-making.

## Outlook

The current consensus [79, 87] is that PGS are not yet ready for widespread implementation in clinical psychiatric practice. Translation will require i) larger, ancestrally diverse GWAS to improve cross-population prediction and enable development of treatment-specific and subtype-specific scores; ii) prospective clinical validation demonstrating outcome improvements; iii) appropriate infrastructure, including decision support tools, standardised reporting, and electronic health record integration; and iv) workforce training in genomic medicine and an expansion of psychiatric genetic counselling.

Last year, the PGC published multiple hallmark studies [15, 33, 48], including the largest GWAS meta-analyses to date for depression and bipolar disorder. These large-scale, multi-ancestry studies will pave the way for several secondary analyses, including PGS analyses. The potential value of any PGS in a specific clinical situation requires assessment in research studies prior to implementation. To improve the accuracy of risk assessment, PGS should be combined with other known risk factors within an integrated model since i) PGS explain only a fraction of the genetic contribution to risk; and ii) genetic factors account for only a fraction of the absolute risk [79, 87].

For all potential applications, the predictive ability of PGS must be improved. This can be achieved, amongst others, by conducting GWAS meta-analyses as well as copy number variant and sequencing studies in larger samples to identify additional common and rare disease-associated variants that can be included in future PGS [99]. Predictive ability could also be improved by the inclusion of further omics data. For example, methylation profile scores (MPS) can be calculated based on epigenome-wide association studies in order to determine individual epigenetic (risk) profiles. Given that DNA methylation is influenced by both genetic and environmental factors, these MPS have the potential to improve prediction, e.g. in combination with PGS [100].

In addition to research into disease development, analysis of the molecular basis of resilience to psychiatric conditions is crucial. Resilience refers to the ability to adapt to stress while maintaining a healthy physical and mental state [101]. For example, a study compared controls with high polygenic risk for schizophrenia with risk-matched schizophrenia patients to develop a polygenic resilience score [102]. Interestingly, an analysis of UK Biobank data showed that schizophrenia polygenic resilience scores interacted significantly with schizophrenia risk scores and conferred protective effects against various psychiatric and somatic disorders including type 2 diabetes [103].

Finally, GWAS meta-analyses of psychiatric conditions still involve individuals of predominantly European ancestry [104]. To ensure the global applicability of PGS, an important goal is the establishment of larger data sets of individuals of non-European ancestry. Notably, studies have shown that when GWAS from different ancestries are used in their development, PGS performance in independent cohorts improves [104, 105].
